# Awareness on oral cancer among patients attending dental school clinics in Brazil

**DOI:** 10.4317/medoral.23207

**Published:** 2019-12-24

**Authors:** Nathália Sousa do Prado, Roberta Ferreti Bonan, Augusto César Leal da Silva Leonel, urema Freire Lisboa de Castro, Elaine Judite de Amorim Carvalho, Fabiana Moura da Motta Silveira, Danyel Elias da Cruz Perez

**Affiliations:** 1 School of Dentistry, Department of Clinical and Preventive Dentistry, Oral Pathology section, Universidade Federal de Pernambuco, Recife, PE, Brazil; 2 Recife Dental School, Recife, PE, Brazil.

## Abstract

**Background:**

Oral cancer is considered a public health problem worldwide. Dental schools may play an important role in educating patients about oral cancer. This study aimed at evaluating the knowledge of patients attending clinics at two dental schools in Brazil.

**Material and Methods:**

From March 2017 to April 2017, 251 patients who were attending clinics at two dental schools in Recife, Brazil, were included in the study. Patients were contacted in the waiting rooms of the clinic. Each participant completed a self-administered questionnaire, which consists of 21 questions, including socio-demographic and specific information on the disease. Data were analyzed using descriptive statistics, and a chi-square test (with a 5% significance level) was used to assess the correlation between the variables, education and family income and other variables.

**Results:**

Most participants were women (64.9%) with a mean age of 42.72 years. Most participants were knowledgeable about oral cancer and identified tobacco use (48.6%), alcohol consumption (25.1%), and solar radiation (12%) as the primary risk factors for the disease. Only 36.7% of the participants reported having received counselling on oral cancer, of which 18.3% received the information from a dentist. All patients with an income higher than six minimum wages were aware about oral cancer (*p* = 0.001).

**Conclusions:**

These findings emphasize the importance of educational programs in dental schools as well providing integrated services for patients seeking care at school clinics, including population’s awareness on oral cancer.

** Key words:**Dental school, knowledge, oral cancer, oral health education, patient education.

## Introduction

Cancer is a major public health problem, which is responsible for the high morbidity and mortality rates worldwide. According to the World Health Organization (WHO), new cancer cases and cancer-related deaths worldwide are projected to reach approximately 26 million and 17 million by 2030, respectively (1). Globally, it is estimated that 354,864 new cases of oral cancer occurred in 2018. In Brazil, oral cancer represents the 5th most frequent cancer among males, with an estimated 11,200 new cases in men and 3,500 in women in each year of the biennium 2018-2019 (2,3). Among the malignancies that affect the oral cavity, squamous cell carcinoma (SCC) is the most prevalent, accounting for 90–95% of the cases (4).

The most prevalent risk factors for oral cancer are alcohol consumption and tobacco use, which have a synergistic effect (5,6). HPV16, betel and ultraviolet radiation for lip carcinoma are also risk factors for the disease (6). In addition, evidence shows that low intake of fruits and vegeTables may be a contributing etiological factor (7,8). In addition, one of the important factors for the survival of patients with cancer is diagnosis at an early stage (9). Late diagnosis may be a result of patient delay, which is the period between the first detection of a sign or symptom and first contact with a healthcare provider, or a professional delay, which is the period from the first examination by a health professional to the final histological diagnosis of the disease (10).

Early diagnosis of oral cancer includes the patient’s perception of the disease and the professional diagnosis and contributes to a significant improvement in the treatment of the disease and maintaining the patient’s quality of life. Therefore, patients must be aware about oral cancer, its signs and symptoms, and unhealthy lifestyle habits that may contribute to the development of the disease (4).

Dental schools have a formative function and play a fundamental role in educating patients about oral cancer. Thus, this study aimed at evaluating the knowledge about oral cancer among patients treated in the clinics of two dental schools in Recife, Pernambuco, Brazil.

## Material and Methods

From March 2017 to April 2017, a cross-sectional study was performed at the two dental school clinics, from the School of Dentistry, Universidade Federal de Pernambuco (public university), and from the Recife Dental School (private school), which are both located at Recife, Pernambuco, Brazil. Ethical approval was obtained from the Institutional Review Board of the Universidade Federal de Pernambuco (protocol number: 62471016.0000.5208). A non-probabilistic sample included male and female patients who were attending clinics at the two dental schools. These patients were contacted in the waiting rooms of the clinics, and informed consent was obtained from each participant. The inclusion criteria included patients older than 18 years who were attending clinics at dental schools, and were at waiting rooms of the clinics. The patients diagnosed with oral cancer or referred for the evaluation of oral lesions suspected of malignancy were excluded from the study, in order to avoid possible bias.

Each participant was interviewed using a self-administered questionnaire, which consisted of 21 multiple-choice questions, adapted from Joseph *et al*. (5). This instrument contained socio-demographic questions (age, gender, neighborhood, city of residence, marital status, and income) and specific information (knowledge about oral cancer, its clinical characteristics, and risk factors; smoking and alcohol consumption; eating habits; and oral hygiene). In addition, questions that addressed the attitude, knowledge, and perception of the participants towards oral cancer were included. Data were collected and analyzed with descriptive statistics using the Statistical Package for Social Sciences (SPSS) version 20, with the relative and absolute distribution of the answers in each of the questions in the questionnaire. The possible answers to the questions about the risk factors and clinical signs of oral cancer were “yes”, “no”, or “do not know”. Subsequently, the variables "education" and "family income" were correlated with the other variables using the chi-square test. A *p-value* < 0.05 was considered statistically significant.

## Results

A total of 251 patients aged 18–80 years were included in this study, of which 163 (64.9%) were women with a mean age of 42.7 years. The percentage of single patients (49.8%) was higher than that of married patients (37.8%). With regard to education level, 86 (34.3%), 42 (16.7%), and 20 (8.0%) of the participants had completed high school, college, and elementary school, respectively. Most patients were non-smokers (222 – 88.4%) and 144 (57.4%) were not alcohol users (Table 1). The monthly income of 105 (41.8%) patients was three Brazilian minimum wages (1 Brazilian minimum wage = approximately U$250). In addition, 92 (36.7%) participants had a monthly minimum wage and 11 (4.4%) had no fixed income.

Most participants (204 - 81.3%) are aware of oral cancer, and 147 (58.6%) affirmed to know the risk factors for the disease. However, when the options of risk factors were listed, of the 251 participants, 122 (48.6%), 63 (25.1%), and 30 (12.0%) identified tobacco use, alcohol consumption, and ultraviolet radiation as the risk factors for oral cancer, respectively. One hundred and four (41.4%) patients pointed unaware of the risk factors for oral cancer. Moreover, other risk factors were indicated by the participants, such as infectious diseases (96 – 38.3%), removable dentures (38 – 15.1%), illegal drug use (32 – 12.8%), cell phone use (14 – 5.6%), obesity (12 – 4.8%), and consumption of spicy food (9 – 3.6%) (Table 2).

**Table 1:**
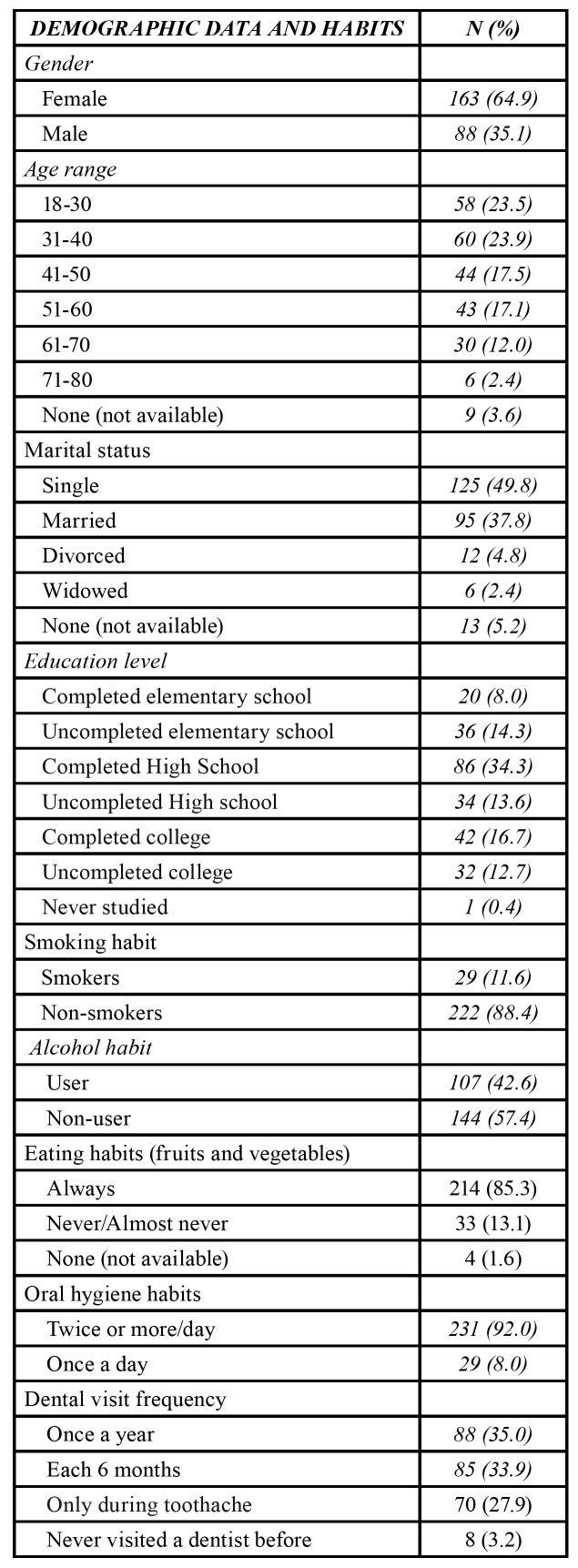
Demographic data and habits of the patients.

**Table 2:**
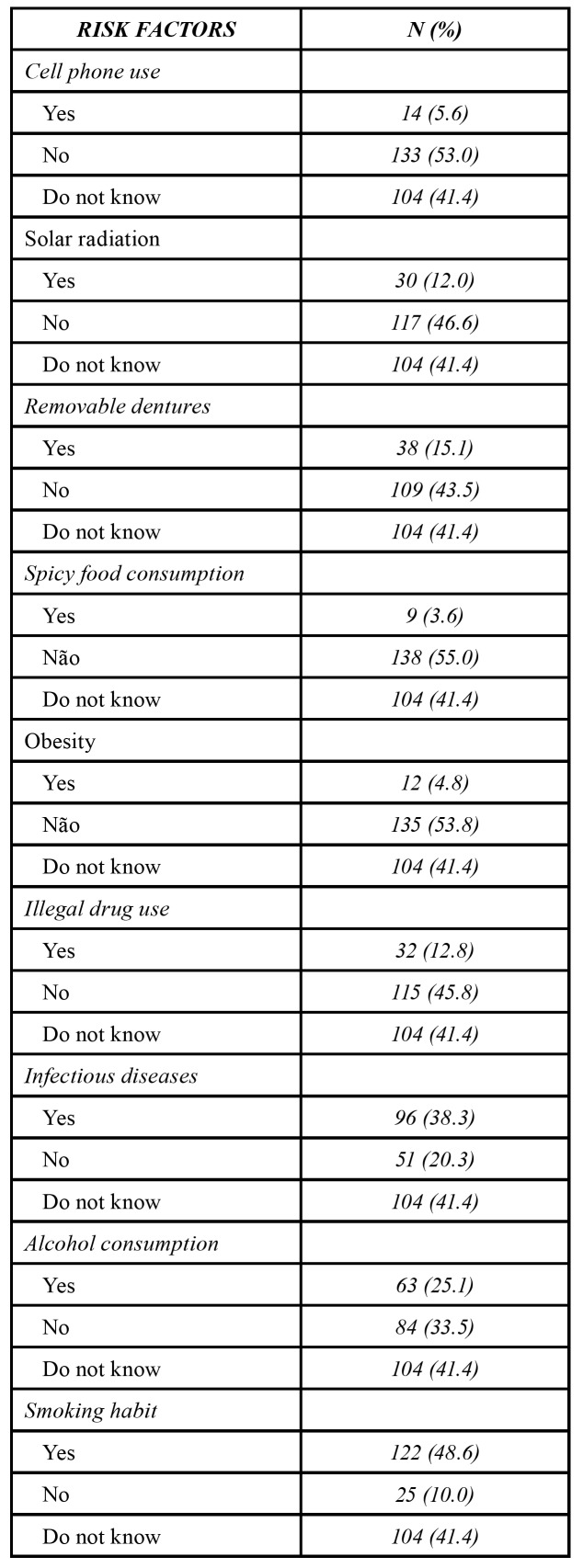
Knowledge on risk factors for oral cancer.

Among the patients, 100 (39.8%) had a family history of cancer. With regard to smoking, 178 (71.7%) denied the habit, 41 (16.3%) had a history of smoking, and 29 (11.6%) are current smokers. In terms of alcohol consumption, 107 (42.6%) participants are current drinkers (Table 1), 27 (10.8%) of them were frequent drinkers, and 80 (31.9%) were occasional drinkers. In addition, 214 (85.3%) participants had a high consumption of fruits and vegeTables.

Only 92 (36.7%) participants received prior education on oral cancer. Of these, 56 (60.8%) stated that they learned from public media (radio, newspaper, internet, and magazines). Only 46 (50%) had received counselling from a dentist, 19 (20.6%) from a physician, and 2 (2.2%) from a pharmacist. Considering all participants (251), only 18.3% received counselling from a dentist and 7.6% from a physician. Less than half of the patients are knowledgeable about the signs of oral cancer, such as white patches (83 - 33.1%), red patches (79 - 31.5%), and nodules in the neck (88 - 35.1%). However, 209 (83.3%) participants were able to identify that a non-healing painless oral ulcer could be a sign of the disease.

In terms of the frequency of dental visits, 85 (33.9%) reported visiting a dentist every 6 months, 88 (35.1%) once a year, and 70 (27.9%) only when they were in pain. However, 8 (3.2%) never visited a dentist. Most of the participants (186 - 74.1%) consulted a dentist less than a year ago. With regard to the frequency of tooth brushing, 231 (92%) participants brush their teeth two or more times per day (Table 1).

In all questions, there was no difference in the response pattern in the two dental schools studied. Statistical analysis revealed that patients with higher and lower education level would contact a dentist when oral cancer is suspected (*p* = 0.047). In addition, all patients with an income higher than six minimum wages are aware about oral cancer (*p* = 0.001) (Table 3). No association was observed between income and risk factors as well as knowledge on clinical signs of oral cancer (Table 4). Moreover, educational level was not correlated with the same variables. However, a statistically significant association between educational level and family income was observed. That is, participants with higher income had higher education level (*p* <0.001).

**Table 3:**
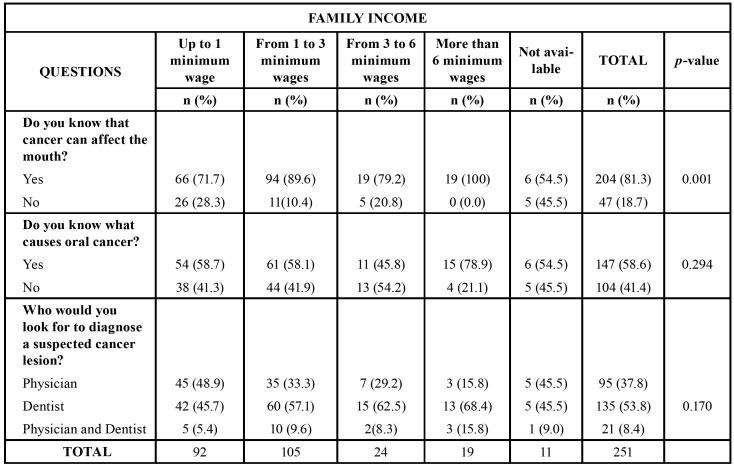
Association between family income and knowledge about oral cancer.

**Table 4:**
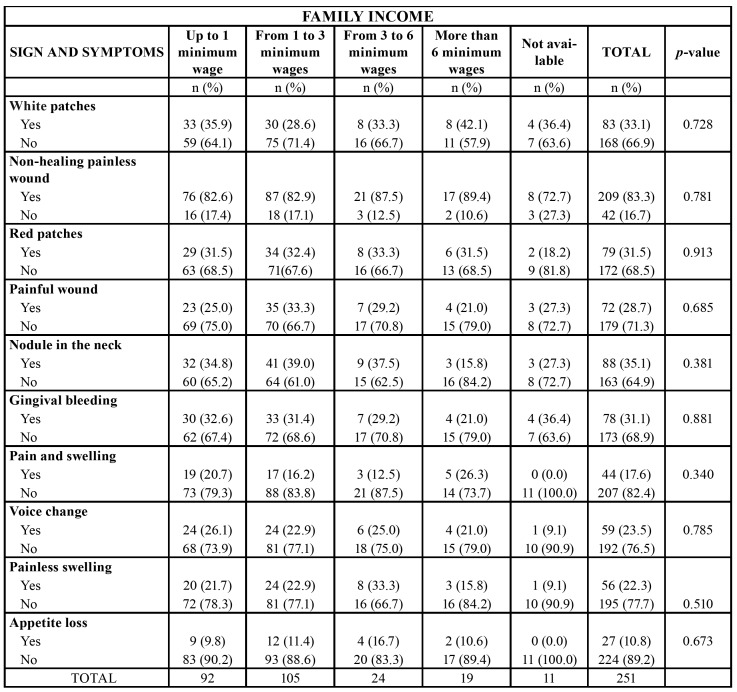
Association between knowledge on signs and symptoms of oral cancer and family income.

## Discussion

The incidence and mortality rates of oral cancer vary according to geographical location. These variations are attributed primarily to cultural and socioeconomic characteristics, environmental factors, education, and health care quality (11). In Brazil, 634,880 new cancer cases are expected in each year of the 2018–2019 biennium, of which 14,700 cases are oral cancer, representing the seventh most common cancer. Despite of this high prevalence in Brazil, data on the level of knowledge on oral cancer are limited. To date, this seems to be the first study that evaluated the awareness on oral cancer among patients attending dental school clinics in Brazil. The present findings may be useful in designing effective education strategies for the patients.

More than 50% of oral cancer cases are diagnosed at advanced clinical stages (12). Delay in diagnosis is common and its definition usually includes patient delay, professional delay and treatment delay (13). Among these three types of delay, the patient delay is the most significant for total delay in diagnosis of oral cancer (14). Public awareness about oral cancer, its risk factors, and signs and symptoms can lead to early diagnosis. Thus, public awareness can result in diagnosis at an early stage, thus increasing the survival rate of patients (11,15). Oral cancer awareness was found to be a significant predictor factor for patient delay (4). Although the lack of public knowledge has been considered a significant barrier for the early diagnosis of oral cancer, this association is not fully elucidated (16).

In similar studies evaluating dental patients, the percentage of participants unaware on the risk factors for oral cancer varies according to the study, from 28.1% (17), 37% (18) to 83.1% (19). In this survey, 41.4% of the patients were unaware of the risk factors. Increased knowledge about tobacco use as a risk factor for oral cancer compared to other factors should be attributed to the anti-tobacco campaigns (12). However, the perception of the patients about the main risk factors remains weak, considering the importance of tobacco use and alcohol consumption in oral carcinogenesis (17,19,20), similar to observed in this survey. In contrast, other studies found a high percentage of patients aware on tobacco and alcohol as important risk factors for oral cancer (5,21). Additionally, in the current study, patients were highly aware that tobacco use is a risk factor for the disease compared to alcohol consumption, and this result is in accordance with previous studies (17,20,21). Only 12% of the participants identified ultraviolet light as an oral carcinogenic. This result reflects the patients’ lack of awareness about this risk factor, or they would not consider the lower lip as one of the possible sites for oral cancer. This study was conducted in a coastal city with a high index of ultraviolet radiation, which makes this result even more alarming. In general, low population awareness about the causal factors of oral cancer reduces the chances of preventing unhealthy lifestyle habits or even contribute to patients’ exposure to carcinogenic agents. Moreover, some patients wrongly pointed to cell phone use, spicy food and obesity as risk factors for oral cancer.

Knowledge on the signs of oral cancer is remarkably unsatisfactory. Considering the main clinical signs of the disease, white/red patches and non-healing ulcer, most patients did not identify these signs as suspected for oral cancer (5,17-19). A study found that 90% of the patients did not know the major signs of the disease (19). In the current study, despite of most patients have recognized a non-healing ulcer as a sign of oral cancer, a low percentage identified white/red patches as oral lesions suspected of malignancy. Moreover, lack of knowledge about the early signs of potentially malignant disorders may result in diagnosis at an advanced stage, and consequently, treatments may be delayed. On the other hand, a study observed that more than 70% of the patients recognized the main clinical signs of oral malignancy (21). In general, the knowledge on oral cancer is proportional to the education level of the patients (15,18,22,23). In the present study, there was association between knowledge about oral cancer and family income, which was significantly associated with educational level. No previous studies found this association between family income and level of awareness on mouth cancer. Other surveys observed different associations. Hassona *et al*. (17) observed that alcohol drinkers and smokers were less informed on signs and symptoms of oral cancer and Villa *et al*. (21) found association between family history of oral cancer and knowledge on risk factors.

In this study, 81% of the patients had hear about oral cancer. In similar studies, this percentage varies from 45% (17) to 72% (18). The participation of dentists in the awareness of patients is alarmingly low. In all comparable surveys, less than 20% of patients received oral cancer counseling by a dentist (17,18,21), similar to found in this study. About 39.5% of the participants stated that they learned about oral cancer from the media, and this result emphasizes the importance of public education. Furthermore, these results support studies that recognize the media as a source of information on oral cancer (23,24). Dentists should assume their role as protagonists in oral health, including the guidance of patients on mouth cancer. Considering the current trend, which shows an increasing number of cases of oral cancer in younger and non-smoking patients (25,26), all patients regardless of age should receive education on this subject. The Internet may be also an important source of health information. Thus, dentists, especially specialists in Oral Pathology and Medicine, should participate more actively in social media on the Internet to disseminate reliable and useful information about the disease. Recently, some authors have pointed to the poor quality of information on oral cancer available on the Internet (27). Moreover, dentists play an important role in identification and diagnosis of oral lesions, including the oral cancer. Complete oral examination and knowledge on the risk factors and main signs and symptoms of the disease are essential for early detection and diagnosis (11). Based on these results, education on oral cancer should targ*et al*l individuals across all social classes. The school-based dental clinics should improve the reception and counselling of their patients, regardless of age or exposure to known risk factor. Teachers and students should reinforce to patients that oral diseases are not restricted to caries and periodontal disease. Patients attending dental school clinics must be educated about oral cancer.

Most of the participants reported that they would seek a dentist in case of suspected oral cancer. However, this finding may be biased because the study was conducted in the waiting rooms of dental school clinics. According to previous studies, more than 60% of the participants would consult a physician, and only 25% would consult a dentist if they had a painful oral ulcer lasting more than 3 weeks (19,28). The literature shows a direct association between a regular visit to the dentist and head and neck cancer (29). Only 33.9% of the participants consulted a dentist every 6 months. Regular dental visits may indicate oral health care and provide more frequent education on the causes and symptoms of oral cancer, in addition to the diagnosis of potentially malignant disorders or oral cancer at an early stage (29).

In summary, patients attending dental school clinics did not have adequate knowledge about the risk factors and primary signs and symptoms of oral cancer. The results emphasize the importance of educational programs in dental schools as well providing integrated services for patients seeking care at these clinics. The data also showed the need to establish public policies, individual or collective, which increase the population’s awareness on oral cancer.
